# Theory of Inverse Edelstein Effect of The Surface States of A Topological Insulator

**DOI:** 10.1038/s41598-017-03346-z

**Published:** 2017-06-16

**Authors:** H. Geng, W. Luo, W. Y. Deng, L. Sheng, R. Shen, D. Y. Xing

**Affiliations:** 1 National Laboratory of Solid State Microstructures and Department of Physics, Nanjing University, Nanjing, 210093 China; 2 Collaborative Innovation Center of Advanced Microstructures, Nanjing University, Nanjing, 210093 China

## Abstract

The surface states of three-dimensional topological insulators possess the unique property of spin-momentum interlocking. This property gives rise to the interesting inverse Edelstein effect (IEE), in which an applied spin bias *μ* is converted to a measurable charge voltage difference *V*. We develop a semiclassical theory for the IEE of the surface states of Bi_2_Se_3_ thin films, which is applicable from the ballistic regime to diffusive regime. We find that the efficiency of the spin-charge conversion, defined as *γ* = *V*/*μ*, exhibits a universal dependence on the ratio between sample size and electron mean free path. The efficiency increases from *γ* = *π*/4 in the ballistic limit to *γ* = *π* in the diffusive limit, suggesting that sufficient strength of impurity scattering is favorable for the IEE.

## Introduction

Spintronics has been a rapidly growing field of research in the past two decades because of its potential applications in memory, logic, and sensing devices, which utilize both spin and charge degrees of freedom of electrons^1–7^. Among the major tasks in spintronics, electrical detection of spin current and spin bias remains to be challenging. One method is to use the inverse spin Hall effect (ISHE), in which a pure spin current generates a measurable transverse charge current^8–11^. While the ISHE has been widely employed in spintronic experiments^12–17^, the electrical signal generated is usually small, e.g., the spin Hall angle ${\theta }_{{\rm{sh}}}\mathrm{=0.08}$ in Pt^18^. Another method that has been attracting increasing interest is the inverse Edelstein effect (IEE)^19, 20^, in which spin injection induces nonequilibrium spin polarization and in turn generates a charge current in the longitudinal direction. The IEE has been observed in Bi^21^, which was attributed to the Rashba spin-orbit coupling on the interface.

Topological insulators (TIs)^22, 23^ and topological Kondo insulators (TKIs)^24^ are a new quantum state of matter. A three-dimensional (3D) TI has a bulk insulating gap with gapless surface states, which are protected from impurity backscattering by nontrivial bulk band topology and time-reversal symmetry. The topological surface states possess the unique property of spin-momentum locking^23–25^, which are promising for applications in spintronic devices^26, 27^. In 2014, large IEE was realized in bulk insulating TIs ${{\rm{Bi}}}_{1.5}{{\rm{Sb}}}_{0.5}{{\rm{Te}}}_{1.7}{{\rm{Se}}}_{1.3}$ and Sn-doped ${{\rm{Bi}}}_{2}{{\rm{Te}}}_{2}{\rm{Se}}$ ^28^, which was interpreted as a result of the spin-momentum locking of the topological surface states. Recently, in another experimental work^29^, the IEE was observed on the surface of TKI ${{\rm{SmB}}}_{6}$. By using a Landauer-Büttiker like formula, Luo *et al*. theoretically studied the IEE of the surface states in the ballistic regime. They predicted that a spin bias polarized in the $y$ direction can generate a charge current flowing in the $x$ direction^30^, which is in agreement with the experimental observation^29^. However, the effect of impurity scattering and sample size dependence in the IEE are not addressed in the simplified theory^30^.

In this work, we employ a semiclassical approach^31^ to study the IEE of the topological surface states. Our analytical theory is applicable from ballistic to diffusive regime. We find that the efficiency $\gamma$ for the spin-charge conversion exhibits a universal dependence on the ratio between sample size ${L}_{x}$ and electron mean free path ${l}_{f}$. When the electron Fermi energy is much larger than the hybridization gap, the efficiency increases from $\gamma =\pi \mathrm{/4}$ in the ballistic limit to $\gamma \mathrm{=1}$ in the diffusive limit, an indication that sufficient strength of impurity scattering is favorable for the IEE. In particular, $\gamma \mathrm{=1}$ corresponds to perfect spin-charge conversion, in which the spin bias is fully converted to an equal amount of charge bias. Our finding may provide an useful guidance for experimental study of the IEE in 3D TIs.

In the next section, we introduce the model equations and the exact solution, and we also present an analytical approximation for the IEE conductance. At the last part of this section the calculated results are discussed. The final section contains a summary.

## Results

### Model and The Exact Solution

Let us start from the effective Hamiltonian of surface states of a thin film of 3D TI ${{\rm{Bi}}}_{2}{{\rm{Se}}}_{3}$ ^32, 33^

1 $$H=\frac{{\rm{\Delta }}}{2}{\hat{\tau }}_{z}{\hat{\sigma }}_{z}+{v}_{f}({p}_{y}{\hat{\sigma }}_{x}-{p}_{x}{\hat{\sigma }}_{y})\mathrm{.}$$

Here, $\vec{p}=({p}_{x},{p}_{y})$ is the electron momentum, ${\hat{\sigma }}_{\alpha }$ with $\alpha =x,y,z$ are the Pauli matrices for electron spin, and ${\hat{\tau }}_{z}$ describes the bonding and antibonding of surface states on the two surfaces, with Δ as the hybridization energy. The eigenenergies for *τ*_*z*_ = ±1 are degenerate, given by

2 $${E}_{{\tau }_{z}}(\vec{p})=\pm \sqrt{{v}_{f}^{2}{p}^{2}+\frac{{{\rm{\Delta }}}^{2}}{4}}\mathrm{.}$$

Here, ${p}^{2}={p}_{x}^{2}+{p}_{y}^{2}$, and signs + and − are for the conduction and valence bands, respectively. The corresponding eigenstates will be denoted as $|{\chi }_{\overrightarrow{p}}^{{\tau }_{z}}\rangle$. The Fermi energy ${E}_{{\rm{F}}}$ is set to be inside the conduction bands. We now calculate the average of ${\hat{\sigma }}_{y}$ in the eigenstates $|{\chi }_{\overrightarrow{p}}^{{\tau }_{z}}\rangle$ by using the Feynman-Hellman Theorem, yieding $\langle {\chi }_{\overrightarrow{p}}^{{\tau }_{z}}|{\hat{\sigma }}_{y}|{\chi }_{\overrightarrow{p}}^{{\tau }_{z}}\rangle =-{v}_{x}/{v}_{f}$ with ${v}_{x}=\partial {E}_{{\tau }_{z}}/\partial {p}_{x}={v}_{f}^{2}{p}_{x}/{E}_{{\rm{F}}}$, which will be used later. The Fermi velocity, being renormalized by the nonzero hybridization energy, becomes ${v}_{{\rm{F}}}={v}_{f}^{2}{p}_{{\rm{F}}}/{E}_{{\rm{F}}}$.

**Figure 1:**
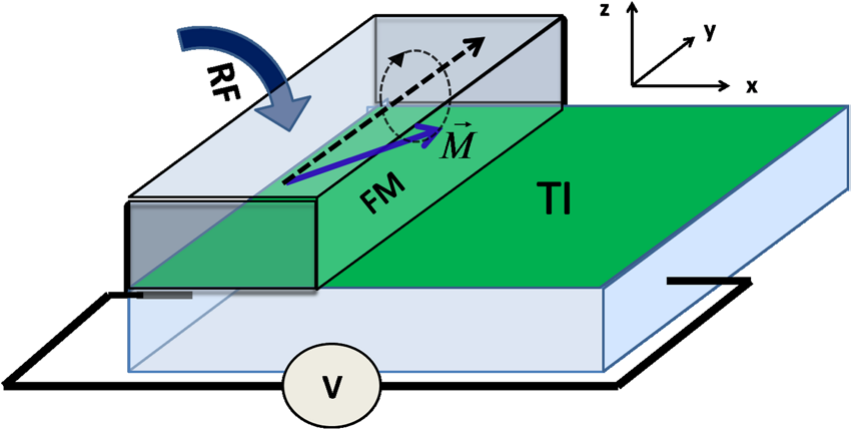
Schematic view of the setup for observing the IEE. A TI thin film is covered partly by a ferromagnetic metal. When the magnetization of the ferromagnet is stimulated to precess around the $y$ axis, by using a radio frequency signal, a spin bias polarized along the $y$ direction will be created in the covered region of the TI film, and electrical current along x-axis will be generated due to the IEE.

Fig. 1 illustrates the setup for observing the IEE. A ferromagnet covers a part of a TI film. When the magnetization is stimulated to precess around a certain direction $\hat{{\bf{n}}}$, a spin bias polarized along $\hat{{\bf{n}}}$ is generated in the covered region of the TI film. In other words, for an electron with spin parallel or antiparallel to $\hat{{\bf{n}}}$, its chemical potential increases or decreases by an amount $(-e\mu )$. The magnitude of spin bias is expected to be proportional to the width of the ferromagnet, as suggested by the previous experiment work on inverse spin Hall effect^17^. The spin bias can be conveniently described by an operator^30^ $(-e\mu )\,\hat{\sigma }\,\cdot \hat{{\bf{n}}}$. In the ballistic regime, it has been demonstrated that for the geometry shown in Fig. 1, only the $y$ component of the spin bias contributes to the IEE effect^30^. Therefore, for simplicity, we focus on the favorable situation, where the spin bias is polarized in the $y$ direction, such that the spin bias becomes $(-e\mu )\,\hat{\sigma }\,\cdot \hat{{\bf{n}}}=(-e\mu ){\hat{\sigma }}_{y}$. The semi-classical boltzmann equation^31^ is used to describe the electronic transport

3 $${v}_{x}\frac{\partial {f}_{{\tau }_{z}}}{\partial x}=-\frac{{f}_{{\tau }_{z}}-{\bar{f}}_{{\tau }_{z}}}{{\tau }_{0}}\mathrm{.}$$

where ${f}_{{\tau }_{z}}(x,{v}_{x})$ is the nonequilibrium distribution function of the electrons in the ${\tau }_{z}$ band, ${\tau }_{0}$ is the relaxation time due to impurity scattering, and ${\bar{f}}_{{\tau }_{z}}$ is the angular average of ${f}_{{\tau }_{z}}(x,{v}_{x})$. As is well-known, the spin diffusion length, which characterizes the spin relaxation process, plays an important role in spin-dependent electronic transport phenomena in conventional metals or semiconductors, where spin relaxation and momentum relaxation are approximately independent processes. The spin diffusion length is usually much longer than the electron mean free path^34^, the latter characterizing the momentum relaxation process. However, owing to the unique property of spin-momentum interlocking of the surface states, spin relaxation is fully coupled with momentum relaxation on a TI surface, and they occur at the same time. As a consequence, both relaxation processes are determined by the electron mean free path on a TI surface. Based on the investigations^36, 37^, the relaxation time of the surface states is about ${\tau }_{0}\simeq 10\,ps$, and the fermi velocity of the surface states is ${v}_{F}\simeq 5.0\times {10}^{5}\,m/s$ ^38^. Therefore, the electron mean free path on the TI surface is ${v}_{F}{\tau }_{0}\simeq 5\,\mu m$. In the linear-response regime, the distribution function takes the form ${f}_{{\tau }_{z}}={f}_{0}+(-\frac{\partial {f}_{0}}{\partial {E}_{{\tau }_{z}}}){g}_{{\tau }_{z}}(x,{v}_{x})$, where ${f}_{0}({E}_{{\tau }_{z}})$ is the equilibrium distribution function. It follows from Eq. 3. that ${g}_{{\tau }_{z}}(x,{v}_{x})$ satisfies the following equation

4 $${v}_{x}\frac{\partial {g}_{{\tau }_{z}}}{\partial x}=-\frac{{g}_{{\tau }_{z}}-{\bar{g}}_{{\tau }_{z}}}{{\tau }_{0}}\,,$$

where ${\bar{g}}_{{\tau }_{z}}(x)=\langle {g}_{{\tau }_{z}}(x,{v}_{x})\rangle \equiv \frac{1}{2\pi }{\int }_{0}^{2\pi }d\varphi {g}_{{\tau }_{z}}(x,{v}_{{\rm{F}}}\,\cos \,\varphi )$ stands for the local angular average of the distribution function.

The region covered by the ferromagnet is treated as a reservoir, and the uncovered region is considered as the sample region. The electron transfer process across the $x\mathrm{=0}$ boundary between the reservoir and sample region is described within the semiclassical approximation^30^. For right-moving electrons, when they just cross the *x* = 0 boundary, their distribution function should still carries the same spin bias as in the reservoir, and thus ${f}_{{\tau }_{z}}(x,{v}_{x}\, > \,0)={f}_{0}+(-\frac{\partial {f}_{0}}{\partial {E}_{{\tau }_{z}}})(-e\mu )\langle {\chi }_{\overrightarrow{p}}^{{\tau }_{z}}|{\hat{\sigma }}_{y}|{\chi }_{\overrightarrow{p}}^{{\tau }_{z}}\rangle$. Here, because $|{\chi }_{\overrightarrow{p}}^{{\tau }_{z}}\rangle$ is the only allowable spin state at momentum $\vec{p}$ in the ${\tau }_{z}$ conduction band that can propagate through the sample region, the spin bias needs to be projected into the subspace spanned by $|{\chi }_{\overrightarrow{p}}^{{\tau }_{z}}\rangle$. Such a projection process accounts for the property of spin-momentum locking of the surface states, which gives rise to the IEE^30^. As a consequence,

5 $$\begin{array}{rcl}{g}_{{\tau }_{z}}(x=\mathrm{0,}{v}_{x} > 0) & = & (-e\mu )\langle {\chi }_{\overrightarrow{p}}^{{\tau }_{z}}|{\hat{\sigma }}_{y}|{\chi }_{\overrightarrow{p}}^{{\tau }_{z}}\rangle \\  & = & e\mu \frac{{v}_{x}}{{v}_{{\rm{f}}}}\mathrm{.}\end{array}$$

The right end of the sample region at $x={L}_{x}$ is assumed to connect to another equilibrium reservoir. When left-moving electrons cross the boundary $x={L}_{x}$, their distribution function remains to be in the equilibrium state, such that

6 $${g}_{{\tau }_{z}}(x={L}_{x},{v}_{x} < 0)=0.$$

Integrating the first-order linear differential equation (4) and taking the boundary conditions Eqs (5) and (6) into consideration, it is easy to obtain a formal solution for the distribution function

7 $${g}_{{\tau }_{z}}(x,{v}_{x})=\theta ({v}_{x})(e\mu \frac{{v}_{x}}{{v}_{{\rm{f}}}}{e}^{-\frac{x}{{v}_{x}{\tau }_{0}}}+{\int }_{0}^{x}{\overline{g}}_{{\tau }_{z}}(\xi ){e}^{-\frac{x-\xi }{{v}_{x}{\tau }_{0}}}\frac{d\xi }{{v}_{x}{\tau }_{0}})+\theta (-{v}_{x}){\int }_{{L}_{x}}^{x}{\overline{g}}_{{\tau }_{z}}(\xi ){e}^{-\frac{x-\xi }{{v}_{x}{\tau }_{0}}}\frac{d\xi }{{v}_{x}{\tau }_{0}},$$

where $\theta ({v}_{x})$ is the unit step function. We note that the unknown function ${\overline{g}}_{{\tau }_{z}}(x)$ appears on the right-hand side, which needs to be solved first. By taking the local angular average on the both sides of Eq. (7), one can derive a self-consistent integral equation for ${\overline{g}}_{{\tau }_{z}}(x)$

8 $${\overline{g}}_{{\tau }_{z}}(x)=e\mu \langle \theta ({v}_{x})\frac{{v}_{x}}{{v}_{{\rm{f}}}}{e}^{-\frac{x}{{v}_{x}{\tau }_{0}}}\rangle +{\int }_{0}^{x}{\overline{g}}_{{\tau }_{z}}(\xi )\langle \frac{\theta ({v}_{x})}{{v}_{x}{\tau }_{0}}{e}^{-\frac{x-\xi }{{v}_{x}{\tau }_{0}}}\rangle d\xi +{\int }_{{L}_{x}}^{x}{\overline{g}}_{{\tau }_{z}}(\xi )\langle \frac{\theta (-{v}_{x})}{{v}_{x}{\tau }_{0}}{e}^{-\frac{x-\xi }{{v}_{x}{\tau }_{0}}}\rangle d\xi \mathrm{.}$$

which can be solved numerically^31^. The local angular average $\langle \cdots \rangle$ is defined below Eq. (4). Equations (7) and (8) constitute the exact solution of the present model. Once ${\overline{g}}_{{\tau }_{z}}(x)$ is obtained, the distribution function can be calculated by using Eq. (7). The electrical current is consequently given by

9 $$I(x)=\frac{e{L}_{y}}{{h}^{2}}\sum _{{\tau }_{z}}\int {v}_{x}{g}_{{\tau }_{z}}(x,{v}_{x})(-\frac{\partial {f}_{0}}{\partial {E}_{{\tau }_{z}}})d{p}_{x}d{p}_{y}\mathrm{.}$$

### An Analytical Approximation

We point out that $\overline{g}(x)$ is essentially the relative change in the chemical potential in the nonequilibrium state, which is a slowly-varying function of position $x$. In ref. 31, it is demonstrated that a linear approximation ${\bar{g}}_{{\tau }_{z}}(x)=a+bx$ to ${\overline{g}}_{{\tau }_{z}}(x)$ generally works very well. In particular, the linear approximation becomes exact in the ballistic limit and diffusive limit^31^. Substituting ${\bar{g}}_{{\tau }_{z}}(x)=a+bx$ into Eq. (8), and choose two arbitrary values of $x$, one can determine the coefficients $a$ and $b$. Following ref. 31, we choose two infinitely close points $x=\frac{{L}_{x}}{2}$ and $\frac{{L}_{x}}{2}+{0}^{+}$ near the middle of the sample region, where the linear approximation is found to be most accurate. We obtain

10 $$a=\frac{{U}_{{\rm{L}}}({L}_{x}+\kappa {l}_{f})}{{L}_{x}+2\kappa {l}_{f}},$$

11 $$b=-\frac{{U}_{{\rm{L}}}}{{L}_{x}+2\kappa {l}_{f}},$$

where ${l}_{f}={v}_{{\rm{F}}}{\tau }_{0}$ is the electron mean free path, ${U}_{L}=(e\mu )\eta \sqrt{1-{{\rm{\Delta }}}^{2}\mathrm{/4}{E}_{{\rm{F}}}^{2}}$, and

12 $$\eta =\frac{{\int }_{-\pi \mathrm{/2}}^{\pi \mathrm{/2}}\,\cos \,\varphi {e}^{-\frac{{L}_{x}}{2{l}_{f}\cos \varphi }}d\varphi }{{\int }_{-\pi \mathrm{/2}}^{\pi \mathrm{/2}}{e}^{-\frac{{L}_{x}}{2{l}_{f}\cos \varphi }}d\varphi },$$

13 $$\kappa =\frac{{\int }_{-\pi \mathrm{/2}}^{\pi \mathrm{/2}}{e}^{-\frac{{L}_{x}}{2{l}_{f}\cos \varphi }}d\varphi }{{\int }_{-\pi \mathrm{/2}}^{\pi \mathrm{/2}}\frac{1}{\cos \,\varphi }{e}^{-\frac{{L}_{x}}{2{l}_{f}\cos \varphi }}d\varphi }\mathrm{.}$$

**Figure 2:**
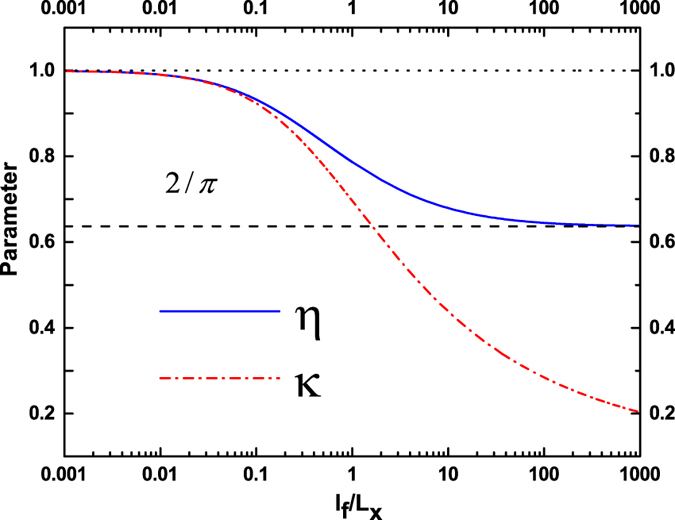
Parameters $\eta$ and $\kappa$ as functions of ${l}_{f}/{L}_{x}$.

We plot the curves for the two parameters $\eta$ and $\kappa$ given in Eqs (12) and (13) in Fig. 2 for reference. We can see that in the ballistic limit ${L}_{x}\ll {l}_{f}$, $\eta \to \mathrm{2/}\pi$ and $\kappa \to 0$. In the diffusive limit ${L}_{x}\gg {l}_{f}$, $\eta \to 1$ and $\kappa \to 1$. These results can also be derived directly from the expressions Eqs (12) and (13).

Following Shen, Vignale, and Raimondi^20^, we define an IEE conductance ${G}_{{\rm{IEE}}}=I/\mu$. By using the above linear approximation to ${\bar{g}}_{{\tau }_{z}}$, analytical expression for ${G}_{{\rm{IEE}}}$ can be obtained as

14 $${G}_{{\rm{IEE}}}={G}_{{\rm{IEE}}}^{0}({\chi }_{{\rm{IEE}}}^{bal}+{\chi }_{{\rm{IEE}}}^{dif})$$

where ${G}_{{\rm{IEE}}}^{0}={G}_{0}\sqrt{1-{{\rm{\Delta }}}^{2}\mathrm{/4}{E}_{{\rm{F}}}^{2}}$ with ${G}_{0}={N}_{{\rm{ch}}}({e}^{2}/h)$ as the Landauer-Büttiker conductance for clean system and ${N}_{{\rm{ch}}}\mathrm{=4}{p}_{{\rm{F}}}{L}_{y}/h$ the number of conducting channels, and

$${\chi }_{{\rm{IEE}}}^{bal}=\frac{1}{2}{\int }_{-\frac{\pi }{2}}^{\frac{\pi }{2}}(\cos \,\varphi -\frac{\eta {L}_{x}}{{L}_{x}+2\kappa {l}_{f}}){e}^{-\frac{{L}_{x}}{2{l}_{f}\cos \varphi }}\,\cos \,\varphi d\varphi ,$$

$${\chi }_{{\rm{IEE}}}^{dif}=\frac{\eta {l}_{f}}{{L}_{x}+2\kappa {l}_{f}}{\int }_{-\frac{\pi }{2}}^{\frac{\pi }{2}}(1-{e}^{-\frac{{L}_{x}}{2{l}_{f}\cos \varphi }}){\cos }^{2}\varphi d\varphi \mathrm{.}$$

We have divided ${G}_{{\rm{IEE}}}$ into two parts, labeled by superscripts “bal” and “dif”, corresponding to contributions from electron ballistic and diffusive transport processes. In the ballistic limit, where ${L}_{x}\ll {l}_{f}$, it is easy to obtain ${G}_{{\rm{IEE}}}=\frac{\pi }{4}{G}_{{\rm{IEE}}}^{0}$. This result is consistent with that obtained by Luo $et$ $al\mathrm{.}$ ^30^ using the Landauer-Büttiker formula in the ballistic regime in the absence of the contact potential barrier. In the opposite diffusive limit, where ${L}_{x}\gg {l}_{f}$, we have ${G}_{{\rm{IEE}}}=\frac{\pi }{2}\frac{{l}_{f}}{{L}_{x}}{G}_{{\rm{IEE}}}^{0}$, which is essentially a Drude like formula.

## Results and Discussions

**Figure 3:**
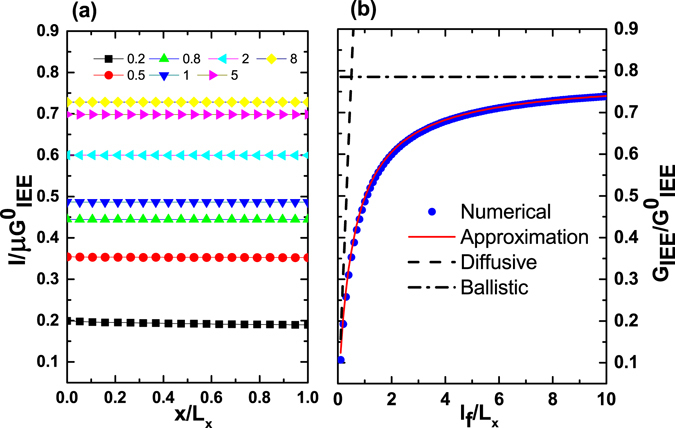
(**a**) Normalized electrical current due to the IEE as a function of normalized coordinate $x/{L}_{x}$ for several different values of ${l}_{f}/{L}_{x}$. (**b**) IEE conductances as functions of ${l}_{f}/{L}_{x}$ calculated from exact numerical solution and approximate formula Eq. (14). The black solid line stands for the result of the Landauer-Büttiker like formula in the ballistic regime, ${G}_{{\rm{IEE}}}/{G}_{{\rm{IEE}}}^{0}=\frac{\pi }{4}$, and the black dashed line stands for the Drude like formula in the diffusive regime, ${G}_{{\rm{IEE}}}/{G}_{{\rm{IEE}}}^{0}=\frac{\pi {l}_{f}}{2{L}_{x}}$.

In Fig. 3(a), we show the exactly calculated electrical current $I(x)$ due to the IEE as a function of position $x$, for several different values of ${l}_{f}/{L}_{x}$. For a given value of ${l}_{f}/{L}_{x}$, $I(x)$ is a constant independent of $x$, meaning that the continuity of the electrical current is satisfied. This serves as an evidence that our numerical result is accurate. In Fig. 3(b), we plot ${G}_{{\rm{IEE}}}/{G}_{{\rm{IEE}}}^{0}$ calculated from the exact solution and approximate formula Eq. (14) as functions of ${l}_{f}/{L}_{x}$. The approximate formula Eq. (14) fits very well with the exact solution.

When the electrical current $I$ flows through the system, it causes a voltage difference $V=I/G$ between the two ends of the system, where $G$ is the electrical conductance of the system. We introduce the ratio $\gamma =V/\mu$ to measure the efficiency of the spin-charge conversion. In general, $\gamma \le 1$, and $\gamma \mathrm{=1}$ would mean perfect spin-charge conversion, in which a spin bias $\mu$ is fully converted to an equal amount of charge bias. Because $I=\mu {G}_{{\rm{IEE}}}$ by definition, the efficiency can also be expressed as $\gamma ={G}_{{\rm{IEE}}}/G$. The expression for $G$ is given by^31^

15 $$G={G}_{0}({\chi }^{bal}+{\chi }^{dif})$$

where

$${\chi }^{bal}=\frac{\kappa {l}_{f}}{{L}_{x}+2\kappa {l}_{f}}{\int }_{-\frac{\pi }{2}}^{\frac{\pi }{2}}{e}^{-\frac{{L}_{x}}{2{l}_{f}\cos \varphi }}\,\cos \,\varphi d\varphi ,$$

$${\chi }^{dif}=\frac{{l}_{f}}{{L}_{x}+2\kappa {l}_{f}}{\int }_{-\frac{\pi }{2}}^{\frac{\pi }{2}}(1-{e}^{-\frac{{L}_{x}}{2{l}_{f}\cos \varphi }}){\cos }^{2}\varphi d\varphi \mathrm{.}$$

Using Eqs (14) and (15), one can calculate the efficiency.

**Figure 4:**
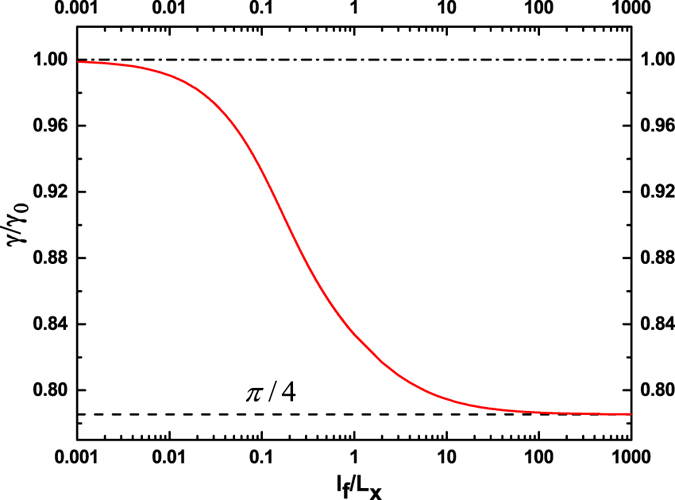
The universal function of $\gamma /{\gamma }_{0}$ versus ${l}_{f}/{L}_{x}$, where ${\gamma }_{0}=\sqrt{1-{\Delta }^{2}\mathrm{/4}{E}_{{\rm{F}}}^{2}}$. $\gamma /{\gamma }_{0}$ approaches $\pi \mathrm{/4}$ in the ballistic limit, and $1$ in the diffusive limit.

It is easy to find that the efficiency $\gamma$ normalized by ${\gamma }_{0}=\sqrt{1-{{\rm{\Delta }}}^{2}\mathrm{/4}{E}_{{\rm{F}}}^{2}}$ is a universal function of ${l}_{f}/{L}_{x}$, independent of any model parameters. The calculated curve of the universal function is displayed in Fig. 4. We see that in the ballistic and diffusive limits, $\gamma /{\gamma }_{0}$ converges to two different constants. In fact, using the expressions for $G$ in the two limits^31^, $G={G}_{0}$ for ${L}_{x}\ll {l}_{f}$, and $G=\frac{\pi }{2}\frac{{l}_{f}}{{L}_{x}}{G}^{0}$ for ${L}_{x}\gg {l}_{f}$, one can readily obtain $\gamma /{\gamma }_{0}=\frac{\pi }{4}$ in the ballistic limit, and $\gamma /{\gamma }_{0}\mathrm{=1}$ in the diffusive limit. We mention that these asymptotic formulas for $\gamma /{\gamma }_{0}$ are exact, because the linear approximation to ${\bar{g}}_{{\tau }_{z}}$ becomes exact in the ballistic and diffusive limits^31^. These asymptotic results can be interpretted as follows. In the ballistic limit, ${L}_{x}\ll {l}_{f}$, all open ${p}_{y}$ channels are equivalent with regard to the conductance, as indicated by the Landauer-Büttiker formula. We can thus replace the right-hand side of the boundary condition Eq. (5) with its average over *p*_*y*_, yielding ${g}_{{\tau }_{z}}(x=\mathrm{0,}{v}_{x} > 0)=\frac{1}{2{p}_{{\rm{F}}}}{\int }_{-{p}_{{\rm{F}}}}^{{p}_{{\rm{F}}}}e\mu \frac{{v}_{x}}{{v}_{f}}d{p}_{y}=$ $e\mu (\frac{{v}_{{\rm{F}}}}{{v}_{f}})\frac{1}{2}{\int }_{-\pi \mathrm{/2}}^{\pi \mathrm{/2}}{\cos }^{2}\varphi d\varphi =e(\frac{\pi }{4}{\gamma }_{0}\mu )$. Therefore, the spin bias *μ* is just equivalent to a charge bias $V=\frac{\pi }{4}{\gamma }_{0}\mu$, and as a result, the efficiency becomes $\gamma =\frac{\pi }{4}{\gamma }_{0}\mu /\mu =\frac{\pi }{4}{\gamma }_{0}$, i.e., $\gamma /{\gamma }_{0}=\frac{\pi }{4}$. In the diffusive limit, ${L}_{x}\gg {l}_{f}$, the electrons incident at small angles with respect to the $x$ axis, i.e., $\varphi \simeq 0$, essentially make dominant contributions to the conductance. For $\varphi \simeq 0$, the boundary condition Eq. (5) reduces to ${g}_{{\tau }_{z}}(x=\mathrm{0,}{v}_{x}\mathrm{ > 0})=e\mu (\frac{{v}_{{\rm{F}}}}{{v}_{f}})\cos \,\varphi =e({\gamma }_{0}\mu )$. The spin bias $\mu$ is equivalent to a charge bias $V={\gamma }_{0}\mu$, and so the efficiency becomes $\gamma ={\gamma }_{0}\mu /\mu ={\gamma }_{0}$, i.e., $\gamma /{\gamma }_{0}\mathrm{=1}$. When the electron Fermi energy ${E}_{{\rm{F}}}$ is much larger than the hybridization gap $\Delta$, we have ${\gamma }_{0}\mathrm{=1}$, so that $\gamma =\frac{\pi }{4}$ in the ballistic limit and $\gamma \mathrm{=1}$ in the diffusive limit. The spin-charge conversion is perfect in the diffusive limit. The perfect conversion efficiency has its origin in the fact that due to spin-momentum interlocking, the spin density and charge current are equivalent for the surface states^35^.

Schwab *et al*. showed that for the topological surface states, the Boltzmann equation should take the form^35^

16 $${v}_{x}\frac{\partial {g}_{{\tau }_{z}}}{\partial x}=-\frac{{g}_{{\tau }_{z}}-{\bar{g}}_{{\tau }_{z}}}{{\tau }_{0}}+\frac{\langle \,\cos \,\theta {g}_{{\tau }_{z}}\rangle }{{\tau }_{0}},$$

where $\theta$ is the relative angle between the two velocities of the incident and outcoming surface states on the Fermi level involved in an scattering event. In comparison with Eq. (4), an additional cosine term appears on the right-hand side of Eq. (16), which accounts for the absence of backscattering due to time-reversal symmetry. This term will increase both the electrical conductance $G$ and IEE conductance ${G}_{{\rm{IEE}}}$, which is interesting and worth to be studied in detail in the future. However, it is expected that this term will not change the efficiency of spin-charge conversion $\gamma ={G}_{{\rm{IEE}}}/G$ dramatically due to cancellation of the numerator and denominator. In particular, according to the above discussion, the spin bias $\mu$ is just equivalent to a charge bias $V=\frac{\pi }{4}{\gamma }_{0}\mu$ in the ballistic limit, ${L}_{x}\ll {l}_{f}$, or $V={\gamma }_{0}\mu$ in the diffusive limit, ${L}_{x}\gg {l}_{f}$. As a result, $\gamma =\frac{\pi }{4}{\gamma }_{0}$ and ${\gamma }_{0}$ in the ballistic and diffusive limits, respectively. This conclusion is drawn without considering the specific electron relaxation mechanism, and so the values of $\gamma$ in the two limits will remain to be exactly the same, even if the absence of backscattering is taken into account. Besides, we mention that disorder violating time-reversal symmetry, such as magnetic impurities, may exist and cause backscattering in actual materials.

## Conclusion

In summary, we have shown that highly efficient IEE or spin-charge conversion can be achieved on a TI surface because of the spin-momentum interlocking of the surface states. An analytical theory for the IEE is developed, which is valid from the ballistic to diffusive regime. The IEE will be very useful for electrical detection of spin current and spin accumulation in spintronics.
